# Healthcare Professionals' Perspectives on Barriers to Reproductive Care Access in One Urban City: A Qualitative Study

**DOI:** 10.1002/nur.70082

**Published:** 2026-05-19

**Authors:** Roxanne Mirabal‐Beltran, Erika Ventura Castellon, Laura Spagna, Kelly Zhang, Karyn J. Roberts

**Affiliations:** ^1^ Georgetown University Berkley School of Nursing Washington District of Columbia USA; ^2^ University of Maryland Baltimore Maryland USA; ^3^ Johns Hopkins University School of Nursing Baltimore Maryland USA; ^4^ Carey Business School Baltimore Maryland USA; ^5^ Georgetown University School of Medicine Washington District of Columbia USA; ^6^ University of North Carolina at Greensboro North Carolina USA

**Keywords:** barriers to care, health care delivery, health equity, qualitative research, women's health services

## Abstract

Inequities exist in reproductive care access for biological women of childbearing age from minoritized, racialized, and lower socioeconomic communities in the United States. While patient‐reported barriers are well documented, less is known about healthcare providers' perceptions of these challenges in urban, under‐resourced contexts. Providers' frontline insights are essential for understanding operational, institutional, and community‐level obstacles and for designing more effective, contextually relevant interventions. This qualitative study explores providers' perspectives on the barriers clients face when accessing reproductive care in Washington, DC. We conducted in‐depth interviews with 14 reproductive healthcare providers. The analysis yielded four themes: (1) limited health literacy: lack of or limited health and resources knowledge, (2) distrust and mistrust: skepticism toward health‐related information and hesitancy to seek reproductive care, (3) challenges navigating care: challenges centered on engagement with available healthcare services, and (4) upstream structural barriers: policy‐driven challenges that affect clinic operations and financial stability. Collectively, these findings underscore persistent barriers to reproductive care, even in contexts with broad insurance coverage, highlighting the disconnect between coverage and meaningful access. Despite high insurance coverage in DC, low‐income, publicly‐insured patients continue to encounter significant obstacles to reproductive care. Addressing these challenges requires policies that ensure healthcare systems provide accessible, patient‐centered, and equitable care, as well as interventions that meaningfully integrate provider perspectives to enhance relevance, feasibility, promote provider engagement and buy‐in, and support the long‐term scalability and sustainability of efforts aimed at improving equitable access.

**Patient or Public Contribution:**

This study provides insight from reproductive healthcare providers serving patients in Wards 1, 4, 5, and 7 of the District of Columbia, Washington. All aspects of this qualitative study were informed by a community‐advisory board (CAB) made up of members from these respective wards.

## 1 Introduction

There are numerous challenges to receiving and providing reproductive health care in the United States (US). Reported barriers include finding clinics that offer essential services or are inclusive of diverse populations and accessing language‐concordant providers. Logistical barriers (transportation, childcare, the ability to take time off work for appointments), as well as policy changes (reductions in Title X family planning clinics), further impede access (Adler et al. 2023). As a result, disparities in reproductive health outcomes persist, particularly among those from minoritized, racialized, and lower socioeconomic backgrounds who experience disproportionate barriers and inequities in care (American College of Obstetricians and Gynecologists [ACOG] 2024; Safarzadeh 2025). While reproductive care includes a range of services, this study focuses on pregnancy and maternal health among biological women of childbearing age.

Social determinants of health (SDOH), such as ethnicity, neighborhoods where people live, socioeconomic status, education, healthcare access, and insurance coverage, account for up to 80%−90% of the modifiable contributors to healthy outcomes, far exceeding the impact of clinical care (Magnan 2017). Insurance coverage has been identified as a critical determinant of reproductive health inequities, disparities, and adverse outcomes (Office of Disease Prevention and Health Promotion n.d.; Sohn 2017). Lack of coverage further limits access to clinic appointments and preventive health services (Sutton et al. 2021). These challenges are interconnected: inadequate coverage reduces patients' ability to afford or secure clinic visits, which limits access to routine screening, infectious disease evaluations, and contraception (Adler et al. 2023).

Nationally, 7.9% of the US population is uninsured (America's Health Rankings 2025). The District of Columbia (DC), however, reports one of the lowest uninsured rates. In 2022, 6.2% of adults 18 and older were uninsured, ranking DC among the top 5.4% of 3145 US cities with the lowest age‐adjusted prevalence of uninsured (Centers for Disease Control [CDC] 2025). Yet, despite relatively high coverage, reproductive health outcomes in DC are not significantly better. Disparities persist when uninsured rates are broken down by SDOH categories such as ethnicity, race, education, and socioeconomic status. For example, while just 2.7% of the non‐institutionalized civilian population is uninsured, Hispanic/Latine (6.7%) and Black/African Americans (3.3%) are disproportionately represented compared to non‐Hispanic Whites (0.8%) (US Census Bureau n.d.). Uninsured rates are also higher among high school graduates (7.9%) compared to those with some college (3.1%) or a higher education degree (1.7%) and among those living at 138%−399% of the poverty threshold (5.2%) compared to those at or above 400% (1.3%) (US Census Bureau n.d.). Among the insured, coverage gaps and high out‐of‐pocket costs persist. A recent study found that Black birthing individuals with commercial insurance faced the highest mean out‐of‐pocket spending, followed by Hispanic, Asian, and White patients, even after adjusting for health and demographic characteristics (Gourevitch et al. 2025).

The 2025 March of Dimes Report Card highlights worsening maternal and infant health indicators in DC, relative to the Healthy People 2030 goals (March of Dimes 2025; Office of Disease Prevention and Health Promotion 2020). Findings include increased rates of preterm births (11.8%; 45th of 52 in the US), infant mortality (7.0 per 1000 births; 43rd of 52), low‐risk cesarean births (31.6%; 51st of 52), severe maternal morbidity (132.8 per 10,000 hospital births; 46th of 47), and maternal mortality (28.2 per 100,000 births; 32nd of 48). DC ranks 51st of 52 in the US in the provision of adequate prenatal care. Black mothers in DC experience higher rates of inadequate prenatal care and adverse outcomes, while Black infants face mortality rates 1.5 times higher than the DC average (10.1 in 1000 live births, 2021−2023). Preterm birth rates among Black women (14.5%) are 130% higher than DC's best rate and double those of White women (7.0%). Nearly half of all live DC births are covered by Medicaid (45.1%), with Medicaid patients experiencing preterm birth rates (14.7%) nearly twice those of privately insured patients (7.7%) and reporting higher rates of inadequate prenatal care.

DC's distinct geographic and health policy environment presents an opportunity to examine structural and systemic obstacles to reproductive care beyond insurance gaps. DC providers are uniquely positioned to identify challenges that affect low‐income urban populations, including those not addressed by coverage alone. While patient‐reported barriers, such as access, logistical challenges, transportation, affordability, fear of stigma, interpersonal relationships, privacy, and lack of awareness (Adler et al. 2023; Cook and Dickens 2014; Peahl et al. 2022), remain essential, provider perspectives offer frontline insights into institutional and community‐level obstacles. These perspectives are essential for designing interventions that are effective and contextually relevant.

This study identifies barriers that providers perceive biological women of childbearing age face in accessing reproductive health care in DC.

## 2 Materials and Methods

All aspects of this study were informed by a community‐advisory board (CAB) (Mirabal‐Beltran 2024). We used a Husserl‐inspired, descriptive phenomenological approach with in‐depth interviews to explore providers' experiences of clients' barriers to reproductive care in DC, guided by Giorgi's scientific phenomenology (Giorgi 2009; Shorey and Ng 2022). A 32‐item COREQ checklist (Supporting Information S1) guided our methods of reporting (Tong et al. 2007). This study was approved by the Georgetown University Institutional Review Board.

### 2.1 Sample and Recruitment

A purposive quota sampling method ensured representation across diverse clinical settings (academic medical centers, urban hospitals, community‐based, non‐profit clinics) and reproductive health providers—advanced practice nurses (APN), physicians (MD), and registered nurses (RN). Providers were required to hold an active DC APN/MD/RN license, accept public insurance, and provide reproductive care to clients in DC Wards 1, 4, 5, or 7. DC is divided into eight wards (Supporting Information S2: Table); these wards were intentionally selected due to persistent inequities in maternal health outcomes, access to care, and economic disparities. Our CAB and research team identified recruitment sites representing targeted clinical contexts. The PI emailed organizational partners, who disseminated study information. Interested providers reached out to the study team, were screened for eligibility, and scheduled for Zoom interviews between October 2022 and May 2023. Of 17 providers screened, three were unable to participate due to scheduling conflicts. Recruitment continued until data sufficiency.

### 2.2 Data Collection

Before data collection, the team discussed the study's aim to enhance positionality awareness and conduct bracketing (Bevan 2014; Northall and Chang 2020; Sundler et al. 2019). An experienced research assistant (RA) and the PI conducted 30–90‐min in‐depth interviews, which were recorded, transcribed, and verified for accuracy. After an introduction, participants gave verbal consent.

The interview guide covered expectations, participant descriptors (occupation, years, and place of practice), and experiences with client barriers to reproductive care in DC, including probes from prior interviews. A post‐interview form was completed to ensure dependability, reflexivity, and document issues. The PI reviewed recordings and memos to assess interview dynamics and information power, guiding identification of emerging patterns (Korstjens and Moser 2018). Data collection stopped when additional interviews no longer yielded new themes, indicating data sufficiency (LaDonna et al. 2021; Malterud et al. 2016). Guided by the concept of informational power (Malterud et al. 2016), a smaller sample was adequate given the study's narrow aim, sample specificity and richness, and participants' relevance to the phenomenon. Each participant received a $75 cash card as compensation.

### 2.3 Analysis

Descriptive statistics on demographics were performed using STATA/SE 17.0 (StataCorp 2017). We adapted Colaizzi's descriptive phenomenological analysis for qualitative interview data to enable systematic extraction of themes (Morrow et al. 2015; Northall and Chang 2020; Sundler et al. 2019). After transcription, a six‐step analysis method was followed. The PI facilitated training. Team members (E.V.C., L.S., K.R., and R.M.B.) independently reviewed transcripts, extracted relevant phrases, formulated meanings, and resolved discrepancies in weekly meetings to develop an initial codebook (Chinh et al. 2019). Transcripts were uploaded to Dedoose for independent coding and validation (SocioCultural Research Consultants, LLC 2016). Two reviewers conducted a second round to strengthen confirmability and credibility, synthesizing themes for reporting (Flynn and Korcuska 2018). Illustrative quotes were extracted and pseudonymized. Mistrust/distrust‐related concepts were also categorized (Griffith et al. 2021).

**Figure 1:**
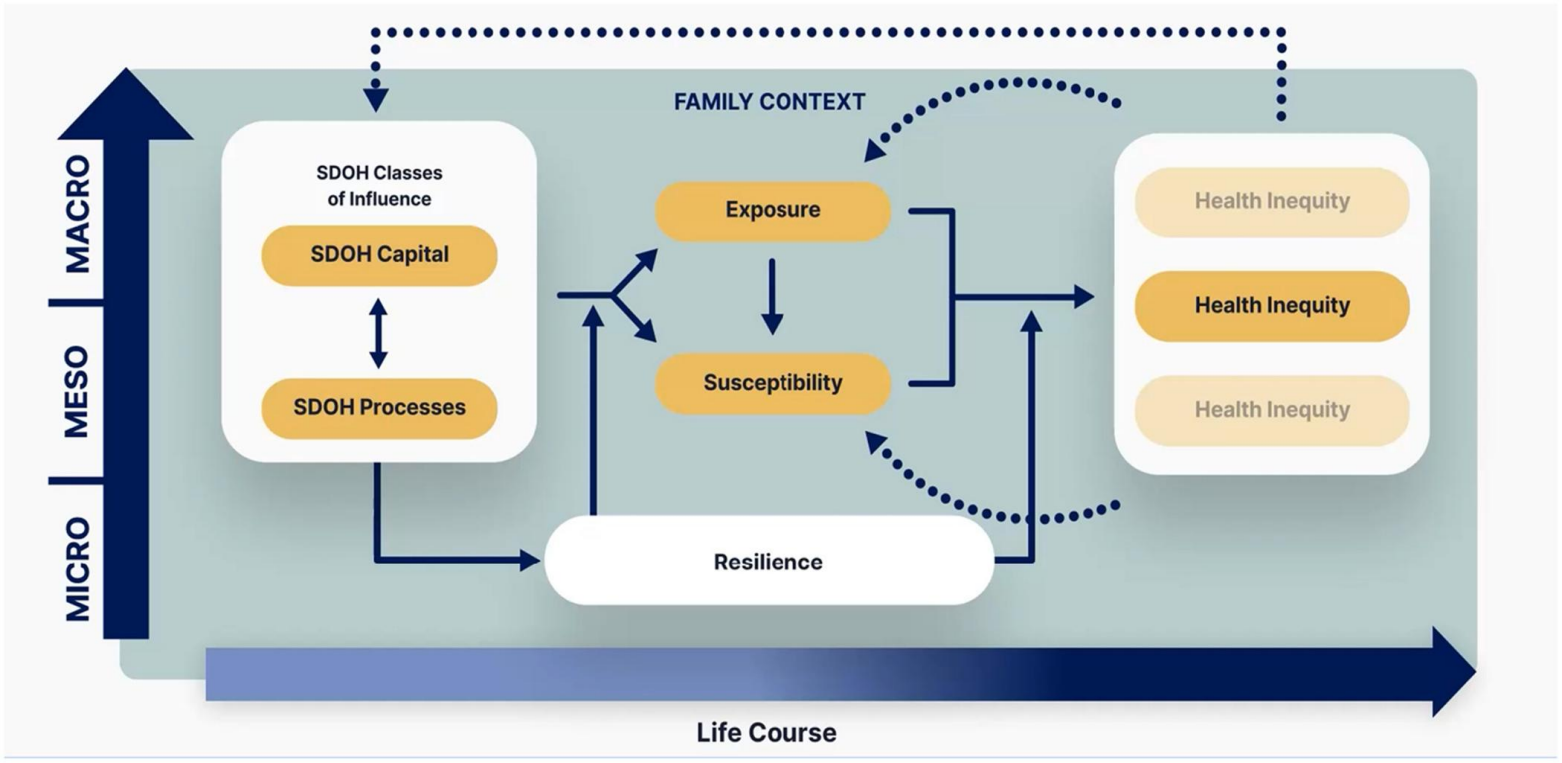
From “Nurse‐led approaches to address social determinants of health and advance health equity: A new framework and its implications,” by Guilamo‐Ramos V, Johnson C, Thimm‐Kaiser M, and Benzekri A. (2023). Nursing Outlook, 71 (6), p. 3 (https://doi.org/10.1016/j.outlook.2023.101996).

Following our thematic analysis, the study team used the Center for Latino Adolescent and Family Health Framework (CLAFH) of SDOH Mechanisms to support interpretation and facilitate translation of qualitative findings into policy‐ and practice‐relevant implications (Figure 1) (Guilamo‐Ramos et al. 2023; Thimm‐Kaiser et al. 2023). CLAFH explains how SDOH operate across macro‐, meso‐, and micro‐levels through capital (health information, access), processes (racism, logistical challenges), risk exposure, susceptibility, and resilience. The PI audited data collection and analysis procedures (Korstjens and Moser 2018). Team assumptions were documented to support reflexivity and bracketing.

## 3 Findings

### 3.1 Participant Characteristics

Fourteen reproductive healthcare providers, comprising four midwives (CNM), four physicians (MD), five nurses (RN), and one nurse practitioner (NP), participated in in‐depth interviews (Table 1). Most providers worked in private practices or hospitals that accept private and public insurance (*n* = 10, 71.4%); four worked in community health clinics. Three providers also worked in home health care, conducting prenatal and postpartum visits in the identified Wards. Participants averaged 17.1 years of clinical experience (SD = 12.3), with experience levels ranging from 1 to 39 years, reflecting variation of experience within the sample.

**Table 1:** Participant characteristics.

Pseudonym^a^	Provider type	Years in practice	Practice type
Angela	Midwife (CNM)	13	Community health clinic
Benji	Midwife (CNM)	9	Community health clinic
Clara	Midwife (CNM)	3	Community health clinic
Daniel	Midwife (CNM)	24	Hospital and private
Emma	Nurse (RN)	24	Hospital
Frank	Nurse (RN)	15	Home health and hospital
Gina	Nurse (RN)	39	Hospital
Humberto	Nurse (RN)	28	Home health and hospital
Isa	Nurse (RN)	1	Hospital
Jorge	Nurse practitioner (NP)	10	Home health and private
Katya	Physician (MD)	20	Community health clinic
Lizette	Physician (MD)	39	Hospital
Mathew	Physician (MD)	8	Hospital
Nestor	Physician (MD)	7	Hospital

^a^ Pseudonyms are used to protect the participants' gender, sex, and identity, ensuring confidentiality.

### 3.2 Qualitative Findings

Reductive analysis yielded four themes capturing the perspectives of providers: limited health literacy, distrust or mistrust, challenges navigating care, and upstream structural barriers.

#### 3.2.1 Limited Health Literacy

A challenge to accessing reproductive care was *health literacy*, defined by providers as literacy related to *health information and available resources*. Providers shared many client experiences related to a *lack of health knowledge or information*. Katya (MD) emphasized that “understanding what a diagnosis might mean, what a condition might mean, what can be done about it, what can't be done about it” are all important for access. Katya further explained: “…there's people coming in with eight complaints and all valid, and you [the provider] want to help them. How can you do all that [give education] in 20 min?” Angela (CNM) stressed that having the correct health information is essential even in the face of other barriers (e.g., access to jobs, childcare, parental leave, health insurance access):

…giving people more information to almost democratize their experience of sitting down with a healthcare provider, that they're armed with a little more information going into some conversations about their healthcare, and that maybe they can make some slightly different choices to be better aligned with their own health desires….

Angela also added that “combating misinformation is as important in a lot of ways as sharing good information…because there are things that people just don't know….”

All providers suggested that clients are often not aware of the *availability of resources* until they have already engaged in the healthcare system. For example, clients are often unaware of insurance covering nurse home visits: “…some of their visits could be having a nurse come to the home…the only way that they [patients] know about that service is there has to be something wrong” (Angela, CNM). While several providers cited lack of transportation as a commonly reported barrier to accessing care, Frank (RN) added that publicly‐insured clients are frequently unaware that coverage includes healthcare‐related transportation services (e.g., Ubers for appointments, hospital visits): “they don't find out until they're already 8 months pregnant…[and then] they're like, ‘I haven't been going because I didn't have a ride.’”

A lack of literacy concerning the *availability of places to access care* was confirmed by providers. Gina (RN) noted that often people end up going to clinics attended by family and friends. If someone does not have those connections, they are unclear of how to find out where they can go:

…there are clinics…but they have to be motivated to find those clinics. Especially ones that take care of patients who don't have any insurance. We have a lot of people in the city who are migrants who have come here, and they don't really know where to look. So, I think a lot of it is just communication about availability.

Gina, along with several other providers, remarked that it was not about a lack of places, but rather a lack of *literacy* concerning what and where those places were. Katya (MD) noted: “There's a lot of resources for under‐resourced populations. But it's just accessing those resources is not the easiest.” With further probing, providers shared that illiteracy concerning where to go may stem from “significant underlying apprehension about dealing with the healthcare system” (Benji, CNM). Mistrust is one reason why another's experiences with the healthcare system becomes a big source of information of where to go. Providers shared the additional challenge that Limited English Proficiency (LEP) presents, and the importance of framing limited literacy within the context of LEP.

Providers were encouraged to share possible solutions to health literacy barriers. Across all types of providers, it was shared that: “Anything that raises people's healthcare IQ is always a benefit…. I don't care where you live, even what your economic status [is]…people just don't know stuff” (Humberto, RN). A critical aspect of raising health literacy was “finding different ways to access people where they're at.” Clinicians are limited to 15 min with patients, and that is “generally not enough time to really sit down and go through a complex topic” (Angela, CNM). Some providers mentioned using perinatal care coordinators and pregnancy apps like Baby Scripts, which “tracks the pregnancy throughout the entire time, gives [the patient] useful information at each trimester or every couple weeks…I think that has improved a lot of education that, like our patients, didn't know beforehand” (Nestor, MD).

#### 3.2.2 Distrust and Mistrust

Providers identified *distrust and mistrust* in the healthcare system as another challenge. Distrust stems from an “…innate sense that the medical community is not supportive of them and that many times hospital settings are places where family members have gone to die” (Lizette, MD). Providers described patients sharing prior negative experiences, including coercion, not having their needs met, or not feeling respected or well served. For this reason, some patients forego seeking care or limit their interactions with health systems. “If folks don't feel safe and supported and like they're receiving care that they can trust, they won't go for care” (Daniel, CNM). Mathew (MD) adds that this distrust is preceded by experiences where “people maybe have not had great encounters in the past, and so they're hesitant to seek care if they think that they're not going to get treated with the respect that they deserve.” Skepticism from the patient perspective, as described by providers, is multipronged. Some patients are hesitant to accept information at face value, especially when providers do not reflect their lived experiences or cultural backgrounds. This skepticism intersects with health literacy, since “…there may be some real hesitation to believing what the providers may say, no matter how devoted the provider may be to the provision of information” (Lizette, MD).

Two participants remarked that there has been an increase in distrust and mistrust since the Covid pandemic.

…a lot more skepticism around physicians and health care in general for the general population than there used to be…all of the back and forth with the Covid information and all that…people are a little more skeptical now…providers need to understand that…you have to gain people trust again… for them to really understand and take what you're saying (Humberto, RN).

Isa (RN) adds that rapid deployment of the Covid vaccine led to increased skepticism about medical recommendations. This presents a unique challenge for reproductive health “…because there's this skepticism in maternal healthcare and then there's this like broader skepticism after the pandemic. You take those together and maternal health is like not set up for success.”

Providers shared the importance of the healthcare system reflecting the community it serves to foster trust. “People like to hear things, especially when it comes to [health] education, from…people that look like them” (Emma, RN). Lizette (MD) added that “trustworthy” sources of patient education must “reflect their own community and people who appear to share their similar values.” Daniel (CNM) remarked on the importance of the patient‐provider relationship.

Patients are best served when they have established a connection with a provider in an environment where they do feel safe, and they have a trusting relationship so that they feel that the information that they're receiving is of high‐quality, that it can be used to make a decision (Daniel, CNM).

Community engagement and outreach by providers were mentioned as strategies for building trust and showing the community “that we're really there to help them live healthy lives” (Nestor, MD). However, this is not always possible when “they're seeing a new person every year or two when they go into a healthcare setting” (Angela, CNM). Providers working at federally‐qualified health centers (FQHC) report high staff turnover at these clinics.

#### 3.2.3 Challenges Navigating Care

Providers of all types mentioned challenges associated with navigating the existing healthcare system. Providers shared that clients are often “just trying to get through the day‐to‐day” (Isa, RN) and balancing *multiple roles and responsibilities*. Katya (MD) shared:

I see women coming into our office with newborns, young children, multiple children, and not having that time to focus on themselves and take care of their healthcare because they are caregivers for everyone else…for their elders, their parents, or other extended family, younger kids.

Providers describe patients deciding between health care recommendations and other priorities. Lizette (MD) shared:

I had a patient who needed to come for fetal monitoring because her baby was small, but it was the choice of either coming for fetal monitoring for that baby or picking up her small child from school. She chose that small child because the baby was inside her still.

Balancing personal healthcare and childcare was a common barrier mentioned by all providers. Patients' access to care was limited due to the few appointments before and after dropping off or picking up children at childcare or school. Providers acknowledged that clinics often have regulations against bringing children to appointments. Therefore, patients without backup support for childcare are unable to attend appointments: “…they'll miss appointments due to transportation issues or not being able to get childcare…. I think that was like a pretty big limiting factor” (Isa, RN).

Providers described patients as lacking “the mental bandwidth to be thinking long‐term” (Isa, RN) or “the bandwidth to go and do and sign up and register and plan and transport and all the other things” (Angela, CNM). Angela adds: “it's beyond the capacities when your time [is] socially and economically constrained by these social and environmental factors, not to mention, shit's far away.”

Another barrier was *transportation*, specifically coordinating it with childcare and work schedules. Those dependent on public transportation have an increased burden as this “takes a lot of time, a lot of energy” (Nestor, MD). Further, public transportation is unreliable: “There's no way the X2 is running on time. You get three buses in a row and then nothing for an hour” (Angela, CNM), adding to care navigation challenges due to lost time from work:

There are definitely places in all of those wards where people can come for OB care, but they may not be in their neighborhood. …they also don't find it always convenient to go because they may lose work, they may be the sole breadwinner…(Lizette, MD).

Providers identified facilitators for care navigation, highlighting messaging and clients' needs to prioritize family. They emphasized framing self‐care as essential for helping loved ones and giving clients time to consider options based on their priorities: “…that's the way you have to spin it to them, like you know, your children are so important to you, but if you're not healthy, you can't help them” (Humberto, RN). Daniel (CNM) added that allowing time for clients “to think about and review options in the context of their own sort of benefits and risks and alternatives framework based on their priorities” would be a way to best serve them.

#### 3.2.4 Upstream Structural and Systemic Barriers

Providers faced challenges identifying facilitators to navigation of care, possibly due to its intersection with larger upstream structural and systemic barriers embedded within the existing healthcare system.

First, participants shared a lack of private providers accepting public insurance, meaning that publicly‐insured individuals often receive care from FQHCs or emergency rooms.

There used to be a lot more private providers that these patients can see, but… the way health care has changed… patients that have any form of state insurance have less and less providers that will take their insurance (Humberto, RN).

Angela (CNM) notes that FQHCs, however, often lack the funding and workforce that private providers have. Participants attributed workforce deficits to lower rates of Medicaid reimbursement, which leads to high turnover of providers in FQHCs; the impact of high turnover on trust building previously came up under the theme of distrust and mistrust.

A second barrier, particularly for clients who cannot get time off for appointments, was limited clinic hours (i.e., during weekday work hours). Humberto (RN) shared that clinic wait times are unusually long, perhaps due to workplace deficits: “There are a lot of clinics that are available to them…but also there is usually a very long wait when they go to the clinics… they have an appointment at nine, and they might not get home until three pm.” Frank (RN) noted that using hospitals as a source of care rather than healthcare office visits due to hours of operation, urgency, or long wait times compounds upstream structural barriers, particularly when hospitals close in the eastern part of the city.

MDs and CNMs also spoke on time constraints and clinical demands, highlighting the intersection of challenges related to limited time spent with clients and the theme of *health literacy*. Daniel (CNM) remarks:

We have a healthcare delivery system that is based primarily on productivity for providers—how many patients we see—and that, in and of itself, is an incentive for us to see patients very quickly. And seeing a patient quickly does not necessarily lend itself to conversation and supporting decision‐making for patients….

Angela (CNM) shared that she gets 15 min to spend with her clients: “I'm booked however many clients I have in a day, and I have my 15 min to meet complex medical social needs. It's generally not enough time to really sit down and go through a complex topic.” Jorge (RN) adds that clients are not receiving enough information at their visits because “providers are either rushed, or they're just not spending enough time, or they just feel like the patient wouldn't listen anyway, so they're not wasting their time to discuss, you know, whatever the topic is.”

Additionally, participants described *living with low income* as a pervasive systemic factor cutting across all themes, shaping how all other challenges are experienced. They noted that economic and social inequities and underinvestment in “communities where people of color, where black people, migrants, brown people, immigrants live…” combined with limited bandwidth, impact access and are related to “policies that keep certain communities in poverty and underinvest in certain areas” (Angela, CNM).

What lowered economic opportunity looks like is more jobs and less time, and less access to childcare to access things like, oh, I wanted to go to that information session, I wanted to join that support group, I wanted to participate in this outreach activity. But when your time is undervalued at a societal level, and you aren't making enough to live on, there isn't spaciousness in your day‐to‐day life to pursue additional information…. That level of overwhelm that comes with poverty is just incredible.

Navigating public insurance within the context of poverty creates additional challenges: “They don't have the insurance coverage, and they may not even be able to get like any kind of public insurance because it's daunting, because it's hard work to even get like Medicaid, how to sign up for Medicaid, and for WIC, or how to navigate insurances” (Emma, RN).

Participants shared that hospital closures exacerbate existing access barriers to care. They emphasized that closures intersect with the challenges of living with low income, further intensifying difficulties in accessing timely reproductive care. Angela (CNM) explained how the loss of nearby healthcare facilities in DC disproportionately affected patients with limited financial resources, who already were facing burdens. She notes: “[There] is a main east/west route across the city. East? Healthcare desert. West? Wealth, access, and concentrations of healthcare systems.… So, when you look at these east/west, even bus trajectories, and the accessibility of public transportation and its timeliness, these are all massive barriers.”

## 4 Discussion

We examined how reproductive healthcare providers perceive the barriers clients face when accessing reproductive care in DC. Experiences revealed four interrelated themes—limited health literacy, distrust and mistrust, challenges navigating care, and upstream structural and systemic barriers. Finalizing these themes during analysis proved challenging, as they frequently overlapped. The common thread across all themes was the shared experience of living with low income. Contrary to the common view that health insurance can mitigate health inequities, our findings demonstrate that expanded coverage alone does not resolve access challenges.

Prior literature examining healthcare providers' perspectives consistently identifies SDOH as a major barrier to accessing care. Challenges include transportation difficulties, housing instability, and/or financial constraints, which compound one another by limiting patients' ability to attend appointments or access resources (Gregory et al. 2025; Rice et al. 2024). Structural obstacles (shortages of staff, fragmented medical record systems, medication stockouts) further impede care delivery (Hagstrom et al. 2025). Studies focused on the experiences of Black patients living with low income highlight additional challenges navigating healthcare systems and securing comprehensive, continuous care, particularly among those with public or no insurance (Peahl et al. 2022). Our findings align with this literature. Integrated care models, care navigators, telemedicine, and the co‐location of medical and social services were offered as solutions to improve outcomes for vulnerable populations by providers in prior studies (Peahl et al. 2022).

By incorporating provider perspectives, this study extends patient‐reported accounts and offers a more comprehensive view of access barriers. While patients describe transportation, logistical challenges, affordability, stigma, and limited access (Adler et al. 2023; Cook and Dickens 2014; Peahl et al. 2022), providers highlight gaps less visible to patients, including fragmented referral pathways, inconsistent communication about existing resources, and institutional constraints that shape service availability (Gregory et al. 2025). For example, although patients frequently cite transportation as a barrier, providers noted that transportation supports are underutilized due to limited awareness, suggesting failures in organizational literacy rather than a lack of resources (National Institutes of Health, 2026). These findings support a comprehensive definition of health literacy which includes personal health literacy (an ability to comprehend health information) and organizational health literacy (how to find and use health information and resources) (Office of Disease Prevention and Health Promotion 2020; NIH National Institutes of Health 2025). Providers also identified administrative burdens, staffing limitations, and policy restrictions that hinder access but are rarely captured in patient‐centered studies. These insights illustrate how individual experiences are embedded within organizational and system‐level dynamics, highlighting the need for multilevel strategies to address access barriers.

### 4.1 Policy and Practice Implications

Our findings have broad relevance to other specialties and geographic regions. Consistent with prior literature, both patients and providers emphasize individualized care, effective communication, patient‐provider rapport, care coordination, and racial/ethnic concordance (Altman et al. 2020; ACOG 2025; Gregory et al. 2025; Mirabal‐Beltran et al. 2025). However, providers in this study described persistent barriers despite their awareness of patients' needs, indicating that poverty and the difficulties of navigating a complex healthcare system continue to be central challenges embedded within SDOH.

Health literacy interventions, though difficult to quantify financially, may reduce barriers when implemented in community settings where people naturally gather (Mirabal‐Beltran et al. 2024). Delivering support in familiar spaces may reduce patient burdens and enhance their sense of agency. Such interventions include education on health, resources, insurance benefits, and housing access (Leininger et al. 2025). Co‐developing interventions with communities and providers is essential to enhancing the feasibility and long‐term sustainability of these efforts.

Another consideration in developing interventions is situating findings within existing frameworks to ensure appropriate mechanisms are addressed. For this study, we applied the CLAFH framework to interpret our findings (Guilamo‐Ramos et al. 2023). CLAFH was used to situate how provider‐identified barriers operate across macro‐, meso‐, and micro‐levels to support translation into policy‐ and practice‐relevant targets. Limited health literacy as a barrier to reproductive healthcare aligns with the CLAFH concept of capital deficit—insufficient education, restricted access to information, and diminished institutional trust. Patients with LEP face additional challenges, including language barriers with transportation vendors and healthcare providers, which discourage service use and further erode trust.

Providers also reported that patient distrust and mistrust of healthcare—rooted in systemic racism, classism, reproductive coercion, and medical mistreatment—often lead to delayed care or disengagement. Distrust arises from negative experiences, while mistrust reflects broader, less defined skepticism (Griffith et al. 2021). Building trust requires sustained patient‐provider relationships and racial/cultural congruence (Moore et al. 2023; Otte 2022; Smith et al. 2018). Provider cultural humility, effective communication, and SDOH‐informed care can reduce power imbalances and center patient perspectives (Lekas et al. 2020). Within CLAFH, these dynamics reflect mechanisms that shape engagement with care.

Systemic dysfunctions were also reported, with providers noting that patients have to broker their care while juggling caregiving and work. Care coordination is defined by the AHRQ as patient‐ and family‐centered team‐based activities that support patients' navigation through the healthcare system (AHRQ Agency for Healthcare Research and Quality 2024). Effective care coordination accounts for patients' social, educational, and behavioral factors; its absence places additional strain on patients managing their care. Care coordination can facilitate health education, trust building, and safe, efficient transitions of care (AHRQ Agency for Healthcare Research and Quality 2024).

Within CLAFH, barriers such as inflexible schedules, lack of childcare, and unreliable transportation are categorized as meso‐level capital deficits, requiring extra personal effort in a rigid, under‐resourced system where individuals rely on public transportation. Cuts in service and disruptions to routes increase burdens, especially for patients without care navigation support (Micka‐Maloy 2023). Single mothers and Black residents experience longer travel times to hospitals compared to other DC residents (Klumpenhouwer et al. 2021). The absence of patient‐centered care coordination, especially for low‐income and/or patients of color, delays or prevents access to reproductive care and reveals persistent structural inequities beyond insurance coverage (Altman et al. 2020).

Despite high insurance coverage in DC, those relying on public insurance face restricted provider access, underfunded clinics, limited hours, and staffing issues—macro‐level capital constraints described in CLAFH. These barriers contribute to unequal care compared to private insurance (Jung et al. 2023; Lewis et al. 2019). Emphasis on patient volume further undermines trust and health literacy. Transportation barriers, meso‐level processes in CLAFH, also reflect macro‐level inequities exacerbated by gender‐biased infrastructures that limit access to care and economic opportunities (Passman et al. 2024). Together, these intersecting barriers—compounded by low‐income and caregiving demands—illustrate how structural forces outweigh clinical determinants in shaping reproductive health access. Addressing these inequities requires multilevel policy solutions that integrate social, systemic, and structural dimensions of care.

### 4.2 Strengths and Limitations

Study strengths include the use of CLAFH as a translational framework to highlight how SDOH barriers identified by providers cluster across micro‐, meso‐, and macro‐levels, providing a template for identifying policy‐ and practice‐based intervention targets. Conducting interviews without preset themes also yielded rich insights into patient barriers. While the findings may not be generalizable to rural areas and uninsured groups, they offer valuable insights for developing interventions. Although centered on providers' views without direct patient input, our findings align with existing literature.

## 5 Conclusion

Despite high insurance coverage in DC, low‐income, publicly‐insured patients continue to face significant barriers to reproductive care. Addressing challenges requires policies that expand coverage, but importantly, ensure healthcare systems provide accessible, patient‐centered, and equitable care. Efforts must focus on interventions that reduce logistical and systemic obstacles at macro‐, meso, and micro‐levels to improve health outcomes and foster trust among marginalized communities.

## Author Contributions

**Roxanne Mirabal‐Beltran:** conceptualization, methodology, validation, formal analysis, investigation, resources, data curation, writing – original draft, writing – review and editing, supervision, project administration, and funding acquisition. **Erika Ventura Castellon, Laura Spagna**, and **Karyn J. Roberts:** formal analysis, writing – original draft, and writing – review and editing. **Kelly Zhang:** writing – original draft, writing – review and editing.

## Ethics Statement

The institutional IRB approved this study. Informed consent was obtained from all study participants.

## Conflicts of Interest

The authors declare no conflicts of interest.

## Positionality Statements

Erika Ventura Castellon is a Latina and native Washingtonian. She has 5 years of experience in pediatrics and community health nursing. Karyn J. Roberts is a bilingual (English/Russian) cisgender White woman and native English speaker, born and raised in a remote, rural, underserved community in the United States, with 30 years of experience in pediatric and family health nursing. Kelly Zhang is a bilingual (English/Mandarin), first‐generation Chinese‐American born and raised in rural Illinois, hoping to study and improve upon health inequities. Laura Spagna is a bilingual (English/Spanish), cisgender Latina, and native Spanish speaker from a low‐income background raised in South Florida with 4 years of experience in community health nursing. Nandi Dube acknowledges that her identities inform her perspective as a Ghanaian and Malawian‐American cisgender woman and a first‐generation American. Roxanne Mirabal‐Beltran is a bilingual (English/Spanish), Latina, and native Spanish speaker, born and raised in an urban, underserved community in the United States, with over 25 years of experience in reproductive health nursing.

## Supporting information

Supporting File 1

Supporting File 2

## Data Availability

The authors have nothing to report.
