# Supplementary material for: Healthcare Professionals' Perspectives on Barriers to Reproductive Care Access in One Urban City: A Qualitative Study
Source: Res Nurs Health. 2026 May 19;49(5):585–94. doi: 10.1002/nur.70082 (PMC13404278; doi:10.1002/nur.70082)
Supplement: Supplementary file 1 [file NUR-49-585-s001.docx]

**Supplement 2:** Ward characteristics

| **Overall Characteristics**  **(DC Office of Planning, n.d.)** | **Ward 1** | **Ward 2** | **Ward 3** | **Ward 4** | **Ward 5** | **Ward 6** | **Ward 7** | **Ward 8** |
| --- | --- | --- | --- | --- | --- | --- | --- | --- |
| Total Population | 79, 373 | 83, 925 | 82,450 | 85,587 | 88,426 | 79,161 | 90,898 | 86, 509 |
| Race and Ethnicity (%)  Black  Hispanic  White | 22.5  20.0  50.3 | 11.2  13.4  65.4 | 8.7  10.0  72.1 | 46.9  21.0  28.8 | 58.0  9.9  26.1 | 22.3  8.0  61.4 | 82.3  7.0  7.5 | 82.5  3.9  9.7 |
| Bachelor degree or higher (%) | 75.7 | 85.0 | 88.0 | 60.1 | 55.3 | 80.0 | 32.1 | 28.7 |
| Age (yrs, median) | 32.7 | 32.3 | 38.4 | 38.9 | 35.7 | 34.0 | 35.0 | 32.5 |
| Household Income (median) | $126,387 | $118,015 | $133,750 | $128, 210 | $97,814 | $123,638 | $69,109 | $50, 931 |
| Rent Cost (median) | $2,169 | $2,160 | $1,943 | $1,625 | $1,706 | $2,245 | $1,230 | $1,363 |
| Home Owners (%) | 35.7 | 37.8 | 49.9 | 62.2 | 45 | 37.9 | 44 | 22.9 |
| Average Household Size | 1.9 | 1.6 | 2.0 | 2.5 | 2.1 | 1.9 | 2.3 | 2.1 |
| Households without a vehicle (%) | 44.4 | 52.3 | 24.3 | 20.3 | 28.7 | 36.4 | 33.7 | 42.4 |
| Public Transportation (%) | 26.2 | 16.3 | 20.1 | 20.7 | 20.0 | 22.0 | 25.1 | 30.8 |
| Poverty Rate (%) | 11.6 | 12.3 | 7.9 | 8.7 | 14.7 | 11.1 | 22.4 | 26.8 |
| Unemployment Rate (%) | 3.9 | 3.1 | 4.2 | 5.8 | 6.8 | 3.2 | 14.3 | 13.9 |
| **Reproductive Health Characteristics (DC Department of Health, 2025)** |  |  |  |  |  |  |  |  |
| Births in DC (%) | 11.3 | 6.8 | 7.8 | 16.2 | 15.6 | 11.2 | 14.5 | 16.5 |
| Age of Mother at Delivery (yrs, median) | 33.7 | 34.4 | 35.5 | 33.9 | 33.3 | 34.2 | 30.7 | 29.6 |
| Births by Low Weight Status | 9.4 | 8.4 | 8.3 | 7.3 | 8.9 | 9.1 | 14.5 | 17.1 |
| First Prenatal Visit (%)  1st Trimester | 69.6 | 73.2 | 73.5 | 68.2 | 68.7 | 78.6 | 69.7 | 53.7 |
| 2nd Trimester | 23.3 | 19.7 | 22.3 | 26.2 | 23.2 | 18.3 | 25.1 | 34.6 |
| 3rdTrimester | 7.0 | 7.1 | 4.2 | 5.5 | 8.0 | 3.2 | 5.3 | 7.3 |
