# Supplementary material for: Healthcare Professionals’ Perspectives on Barriers to Reproductive Care Access in One Urban City: A Qualitative Study
Source: Res Nurs Health. Author manuscript; Available in PMC 2026 Jul 27. (PMC13404278; doi:10.1002/nur.70082)
Supplement: Supplement 1 [file NIHMS2195192-supplement-Supplement_1.docx]

**Supplement 1 EQUATOR 32-item research checklist: Consolidated criteria for reporting qualitative studies (COREQ)** (Tong et al., 2007)

We used the rigorous 32-item COREQ checklist for standardizing our reporting of methods in this study.

| **Domain** | **Guide Questions** | **Reported** |
| --- | --- | --- |
| **Research Team and Reﬂexivity** |  |  |
| *Personal Characteristics* |  |  |
| Interviewer/facilitator | Which author/s conducted the interview? | Yes |
| Credentials | What were the researcher’s credentials? | Yes |
| Occupation | What was their occupation at the time of the study? | Yes |
| Gender | Was the researcher male or female? | Yes |
| Experience and training | What experience or training did the researcher have? | Yes |
| *Relationship with Participants* |  |  |
| Relationship established | Was a relationship established prior to study commencement? | No |
| Participant Knowledge of Interviewer | What did the participants know about the researcher? | No |
| Interviewer characteristics | What characteristics were reported about the interviewer/facilitator? | No |
| **Study Design** |  |  |
| *Theoretical Framework* |  |  |
| Methodological Orientation | What methodological orientation was stated to underpin the study? | Yes |
| *Participant Selection* |  |  |
| Sampling | How were participants selected? | Yes |
| Method of approach | How were participants approached? | Yes |
| Sample size | How many participants were in the study? | Yes |
| Non-participation | How many people refused to participate or dropped out? Reasons? | Yes |
| *Setting* |  |  |
| Setting of Data Collection | Where was the data collected? e.g. home, clinic, workplace | Yes |
| Presence of Non-Participants | Was anyone else present besides the participants and researchers? | Yes |
| Description of Sample | What are the important characteristics of the sample? | Yes |
| *Data Collection* |  |  |
| Interview Guide | Were questions, prompts, guides provided? Was it pilot tested? | Yes, but it was not pilot tested. |
| Repeat Interviews | Were repeat interviews carried out? If yes, how many? | N/A |
| Audio/Visual Recording | Did the research use audio or visual recording to collect the data? | Yes |
| Field Notes | Were ﬁeld notes made during and/or after the interview? | Yes |
| Duration | What was the duration of the interviews or focus group? | Yes |
| Data Saturation | Was data saturation (sufficiency) discussed? | Yes |
| Transcripts Returned | Were transcripts returned to participants for comment and/or correction? | N/A |
| **Analysis and Findings** |  |  |
| *Data Analysis* |  |  |
| Number of Data Coders | How many data coders coded the data? | Yes |
| Description of the Coding Tree | Did authors provide a description of the coding tree? | No |
| Derivation of Themes | Were themes identiﬁed in advance or derived from the data? | Yes |
| Software | What software, if applicable, was used to manage the data? | Yes |
| Participant Checking | Did participants provide feedback on the ﬁndings? | N/A |
| *Reporting* |  |  |
| Quotations Presented | Were participant quotations presented to illustrate the themes/ﬁndings? Was each quotation identiﬁed? | Yes |
| Data and Findings Consistent | Was there consistency between the data presented and the ﬁndings? | Yes |
| Clarity of Major Themes | Were major themes clearly presented in the ﬁndings? | Yes |
| Clarity of Minor Themes | Is there a description of diverse cases or discussion of minor themes? | Yes |
