# Supplementary material for: Healthcare Professionals’ Perspectives on Barriers to Reproductive Care Access in One Urban City: A Qualitative Study
Source: Res Nurs Health. Author manuscript; Available in PMC 2026 Jul 27. (PMC13404278; doi:10.1002/nur.70082)
Supplement: Supplement 2 [file NIHMS2195192-supplement-Supplement_2.pdf]

Supplement 2: Ward characteristics

**Overall Characteristics (DC Office of Planning, n.d.)**	**Ward 1**	**Ward 2**	**Ward 3**	**Ward 4**	**Ward 5**	**Ward 6**	**Ward 7**	**Ward 8**
Total Population	79, 373	83, 925	82,450	85,587	88,426	79,161	90,898	86, 509
Race and Ethnicity (%)								
Black	22.5	11.2	8.7	46.9	58.0	22.3	82.3	82.5
Hispanic	20.0	13.4	10.0	21.0	9.9	8.0	7.0	3.9
White	50.3	65.4	72.1	28.8	26.1	61.4	7.5	9.7
Bachelor degree or higher (%)	75.7	85.0	88.0	60.1	55.3	80.0	32.1	28.7
Age (yrs, median)	32.7	32.3	38.4	38.9	35.7	34.0	35.0	32.5
Household Income (median)	$126,387	$118,015	$133,750	$128, 210	$97,814	$123,638	$69,109	$50, 931
Rent Cost (median)	$2,169	$2,160	$1,943	$1,625	$1,706	$2,245	$1,230	$1,363
Home Owners (%)	35.7	37.8	49.9	62.2	45	37.9	44	22.9
Average Household Size	1.9	1.6	2.0	2.5	2.1	1.9	2.3	2.1
Households without a vehicle (%)	44.4	52.3	24.3	20.3	28.7	36.4	33.7	42.4
Public Transportation (%)	26.2	16.3	20.1	20.7	20.0	22.0	25.1	30.8
Poverty Rate (%)	11.6	12.3	7.9	8.7	14.7	11.1	22.4	26.8
Unemployment Rate (%)	3.9	3.1	4.2	5.8	6.8	3.2	14.3	13.9
**Reproductive Health Characteristics (DC Department of Health, 2025)**								
Births in DC (%)	11.3	6.8	7.8	16.2	15.6	11.2	14.5	16.5
Age of Mother at Delivery (yrs, median)	33.7	34.4	35.5	33.9	33.3	34.2	30.7	29.6
Births by Low Weight Status	9.4	8.4	8.3	7.3	8.9	9.1	14.5	17.1
First Prenatal Visit (%)								
1st Trimester	69.6	73.2	73.5	68.2	68.7	78.6	69.7	53.7
2nd Trimester	23.3	19.7	22.3	26.2	23.2	18.3	25.1	34.6
3rdTrimester	7.0	7.1	4.2	5.5	8.0	3.2	5.3	7.3
